# Fermented plant product (FPP) suppresses immediate hypersensitivity reactions with impaired high-affinity IgE receptor (FcεRI) signaling

**DOI:** 10.1007/s10616-025-00729-3

**Published:** 2025-02-25

**Authors:** Tomoki Kodama, Ayana Yokoyama, Yuki Nishioka, Riku Kawasaki, Aiko Teshima, Akira Maeda, Ayano Hojo, Takumi Suizu, Hideto Torii, Kotaro Fujioka, Shinsuke Kishida, Takashi Fujimura, Kenji Arakawa, Atsushi Ikeda, Seiji Kawamoto

**Affiliations:** 1 Program of Biotechnology, Graduate School of Integrated Sciences for Life, Hiroshima University, 1-3-1 Kagamiyama, Higashi-Hiroshima, 739-8530 Japan; 2 Hiroshima Research Center for Healthy Aging (HiHA), Hiroshima University, Higashi-Hiroshima, Japan; 3 Program of Applied Chemistry, Graduate School of Advanced Science and Engineering, Hiroshima University, Higashi-Hiroshima, Japan; 4 Manda Fermentation Co. Ltd, Onomichi, Japan

**Keywords:** Allergy, Fermented plant product (FPP), IgE, Mast cells, Passive cutaneous anaphylaxis (PCA)

## Abstract

Fermented plant product (FPP) is a dietary supplement made by fermentation and aging of a variety of plants, including fruits, vegetables, and grains. A previous study has shown that oral FPP supplementation prevents the development of allergic rhinitis-like nasal symptoms in a murine model of Japanese cedar pollinosis without affecting systemic immune response. However,　the mode of action by which FPP exerts an anti-allergic effect remains to be elucidated. Here, we show that FPP acts on mast cells to suppress immediate hypersensitivity reactions in vitro as well as in vivo. We found that stimulation with FPP potently suppressed IgE antibody-mediated degranulation of RBL-2H3 rat basophilic leukemia cells. We also found that oral feeding with FPP significantly suppressed passive cutaneous anaphylaxis (PCA), an in vivo model of IgE- and mast cell-mediated hypersensitivity reactions. Mechanistic analysis revealed that FPP extensively suppressed the high-affinity IgE receptor (FcεRI) signaling pathway, in which FPP not only inhibited intracellular Ca^2+^ influx upon FcεRI ligation but also negatively regulated another Ca^2+^-independent FcεRI signaling pathway leading to granule translocation through microtubule formation. These results suggest that FPP fulfills its anti-allergic activity by acting on the IgE-mast cell axis to suppress immediate hypersensitivity reactions.

## Introduction

The increased prevalence of allergy is a worldwide problem (Weinberg 2011). Typical type I allergy is triggered upon allergen-triggered cross-linking of IgE antibodies that have been pre-sensitized on mast cells (Rajan 2003). This mast cell surface IgE cross-linking induces the release of histamine and other proinflammatory mediators, which in turn leads to immediate hypersensitivity reactions causing atopic symptoms, including asthma, rhinitis, eczema, and food anaphylaxis (Rajan 2003) (Zhang et al. 2023). Symptomatic anti-allergic drugs are designed to antagonize those mediator molecules for relief of the allergic conditions, but current drugs still have side effects reducing the quality of life (Zhang et al. 2023) (Kay 2000) (Malone 2017), and no curative effect is obtained by such symptomatic medications. Allergen-specific immunotherapy (AIT) represents another clinical option for allergy with the hope of curative outcomes, although current therapies still have problems, such as the need for long-term medication, varied therapeutic effects, and the lack of precise therapeutic mechanisms (Akdis and Akdis 2011) (Fujimura and Kawamoto 2015).

In such situations, there is a growing interest in the potential of functional foods with anti-allergic effects as alternatives to allergy medications, aiming for symptom alleviation and preventive benefits (Fujimura et al. 2023). One such example of a functional foodstuff is an edible herb with anti-allergic potency. One of those examples is *Perilla frutescens*, an anti-inflammatory medicinal herb traditionally consumed in East Asian countries (Kamei et al. 2017) (Wu et al. 2023), and responsible anti-allergic compounds have been identified (Kamei et al. 2017) (Wu et al. 2023) (Oh et al. 2011).

In addition to those medicinal plants, fermented foods are also known to provide health benefits, including anti-inflammatory effects (Shahbazi et al. 2021) (Sanlier et al. 2019) (Baruah et al. 2022). Fermented plant product (FPP) is a healthy supplemental food obtained through long-term fermentation and aging (over 3 years) of a variety of fruits, vegetables, grains, and algae (Kawai 1997) (Kawai et al. 1998) (Marotta et al. 2009) (Ashida 2005) (Fujimura et al. 2018). Several health-promoting effects of FPP have been reported in various pathological conditions, such as the alleviation of stomach ulcers (Kawai 1997), inhibition of lipid peroxidation (Kawai et al. 1998), anti-cancer effect (Marotta et al. 2009), and activation of innate immunity (Ashida 2005).

Our research group has shown that oral feeding with FPP prevents the development of allergic rhinitis-like nasal symptoms in a murine model of seasonal pollinosis without affecting allergen-specific adaptive immunity, including T helper type 2 (Th2) response and IgE antibody production (Fujimura et al. 2018). In this system, the oral FPP supplementation tends to decrease the serum level of the in vivo mast cell activation marker, mouse mast cell protease-1 (mMCP-1) (Fujimura et al. 2018), implicating that FPP may act on mast cells to fulfill its anti-allergic effect. Based on this assumption, here we tested whether FPP showed any inhibitory activity on the mast cell-mediated allergic response and further investigated the underlying mechanisms. We show that FPP acts directly on mast cells to suppress IgE-mediated type I hypersensitivity reactions in vitro as well as in vivo. We also provide mechanistic evidence that FPP negatively regulates FcεRI signaling pathway to suppress immediate hypersensitivity reactions.

## Materials and methods

### Fermented plant product (FPP) and preparation of its water-soluble fraction

Fermented plant product (FPP, MANDA^®^) was manufactured and provided by Manda Fermentation Co., Ltd. (Onomichi, Japan). For in vitro experiments, FPP was dissolved in a Modified Tyrode’s buffer [MT buffer; 135 mM NaCl, 5 mM KCl, 2.7 mM CaCl_2_, 1.0 mM MgCl_2_, 5.6 mM D (+)-glucose, and 0.05% bovine serum albumin (BSA) in 20 mM HEPES, pH 7.4], and centrifuged at 15,000 × *g* for 10 min. The resultant water-soluble FPP sample was then sterilized using a DISMIC 0.2 μm filter (ADVANTEC, Tokyo, Japan), and used as for in vitro experiments.

### Antibodies

All antibody reagents were purchased from Cell Signaling Technology (Danvers, MA, USA) as follows; anti-Syk monoclonal antibody (mAb, #13198, 1:1250 dilution), anti-phospho-Syk (pSyk, pTyr 525/526) mAb (#2710, 1:1000), anti-PLCγ1 mAb (#5690, 1:1000), anti-phospho-PLCγ1 (pPLCγ1, pTyr 783) mAb (#2821, 1:1000), anti-PI3 kinase (PI3K) p85 mAb (#4257, 1:1000), anti-phospho-PI3K p85 (pTyr 458)/p55 (pTyr199) antibody (Ab) (#4228, 1:1000), anti-Akt Ab (#9272, 1:2500), anti-phospho-Akt (pAkt, pSer 473) Ab (#9271, 1:1000), anti-GSK3β mAb (#12456, 1:5000), anti-phospho-GSK3β (pGSK3β, pSer 9) mAb (#5558, 1:2500), horseradish peroxidase (HRP)-conjugated anti-rabbit IgG Ab (#7074, 1:2500), and Alexa Fluor^®^ 488-conjugated anti-α-tubulin mAb (#5063, 1:200).

### Cell culture

RBL-2H3 rat basophilic leukemia cells were obtained from the Riken Bioresource Center (RIKEN BRC, Ibaraki, Japan), and cultured in minimum essential medium (MEM; Thermo Fisher Scientific, Waltham, MA, USA) supplemented with 10% fetal bovine serum (FBS; Sigma-Aldrich, St. Louis, MO, USA) and 100 units/ml penicillin–streptomycin (Thermo Fisher Scientific) in humidified 5% CO_2_ atmosphere at 37 °C.

### β-hexosaminidase release assay

RBL-2H3 cells were seeded in 96-well plates at a density of 6 × 10^4^ cells/well, and pre-cultured for 8 h in humidified 5% CO_2_ atmosphere at 37 °C. Cells were sensitized with 50 ng/ml anti-DNP IgE mAb (YAMASA Co., Ltd., Chiba, Japan) diluted in 10% FBS-MEM medium (200 µl/well) and incubated for 16 h. Unsensitized cells were used as a negative control. The IgE-sensitized cells were washed with phosphate-buffered saline (PBS), and treated with FPP (7.5–15 mg/ml) or 500 μM ketotifen fumarate (KF; a positive control inhibitor of degranulation, FUJIFILM Wako Pure Chemical, Osaka, Japan) diluted in MT buffer (vehicle), and incubated for 30 min. Cells were then stimulated with 0.5 μg/ml DNP-BSA antigen (Ag) at 37 °C for 30 min to induce degranulation, and the reaction was stopped for 10 min incubation at 4 °C. For β-hexosaminidase assay, culture supernatant (50 µl/well in a 96-well microtiter plate) was mixed with 5 mM *N*-acetyl-β-D-glucosaminide substrate, and incubated at 37 °C for 30 min, followed by addition of 50 μl glycine buffer (pH 10.4) to stop the enzymatic reaction. The absorbance at 405 nm was then measured by Wallac 1420 ARVOsx Multilabel Counter (PerkinElmer Life Sciences, Waltham, MA, USA), and β-hexosaminidase release (%) was calculated as following formula; β-hexosaminidase release (%) = 100 x [(A_supernatant_)-(A_blank of FPP_)] / [ (A_cell lysate_)-(A_blank of cell lysate_)], in which “A” is the absorbance of each well.

### Cell viability assay

Cell viability was analyzed by trypan blue dye exclusion assay. RBL-2H3 cells were seeded in 24 well plates at a density of 1.5 × 10^5^ cells/well and sensitized with anti-DNP IgE mAb as described above. Cells were then treated with serial concentrations of FPP or 500 μM KF for 30 min, followed by stimulation with Ag for 30 min. After washing with PBS and trypsinization, cell suspension was stained with 0.4% trypan blue dye solution (Thermo Fisher Scientific). The cell viability ratio was calculated as follows; Cell viability (%) = S/T × 100, in which S is the number of unstained cells and T is the total cell number.

### Passive cutaneous anaphylaxis (PCA)

PCA was performed using the previously described method with minor modifications (Kawasaki et al. 2023). Briefly, five-week-old female BALB/c mice (Jackson Laboratory Japan Inc., Kanagawa, Japan) maintained under specific pathogen-free conditions were fed AIN-93N diet containing 5% (w/w) FPP (manufactured by Oriental Yeast Co., Ltd., Tokyo, Japan) or control AIN-93N diet (Oriental Yeast) for 64 days. On day 63, anti-DNP IgE (150 ng, in 10 μl PBS) or PBS (vehicle control) was intradermally injected into the right or left ear of each mouse, respectively. After 24 h, 200 μg DNP-human serum albumin (HSA, Sigma-Aldrich) with 1% Evans blue (EB) dye solution (100 μl) was intravenously injected into their tail vein. After 30 min, the PCA reaction was evaluated based on the amount of auricular extravasation of EB dye. For quantification, the EB dye was extracted by 24 h incubation of ear samples with formamide at 63 °C, and their amounts were quantified by spectrophotometry upon measurement of absorbance at 595 nm. All animal experiments were carried out using protocols (protocol number: C20-2–10) approved by the Committee on Animal Experimentation of Hiroshima University, Japan.

### Measurement of intracellular Ca^2+^ influx

Measurement of intracellular Ca^2+^ influx was performed using the previously described methods (Kamei et al. 2017) (Karakus et al. 2020) (Chen et al. 2017) with minor modifications. For the measurement of IgE-dependent Ca^2+^ influx, anti-DNP IgE mAb-sensitized RBL-2H3 cells were loaded with 5 μg/ml Fluo 4-AM in a loading buffer [1.25 mM probenecid, 0.04% Pluronic®F127 (Dojin Chemical Laboratories, Kumamoto) in MT buffer] at 37 °C for 1 h. After washing with PBS, the cells were treated with FPP (12.5–15 mg/ml) at 37 °C for 30 min, followed by stimulation with 0.5 μg/ml DNP-BSA. Changes in fluorescence intensity were monitored with Wallac 1420 ARVOsx Multilabel Counter (PerkinElmer Life Sciences). For the measurement of store-operated Ca^2+^ entry (SOCE), Fluo 4-AM-loaded RBL-2H3 cells were stimulated with 0.9 μM ionomycin (Nacalai tesque, Kyoto, Japan) and fluorescence was detected as described above. Alternatively, the Fluo 4-labelled RBL-2H3 cells were pre-chelated for 100 s with MT buffer containing 0.5 mM ethylene glycol tetraacetic acid (EGTA, Dojin Chemical Laboratories), and then stimulated with 10 μM thapsigargin (TG, FUJIFILM Wako Pure Chemical) for 200 s to induce the primary Ca^2+^ release from endoplasmic reticulum (ER). Then the secondary SOCE was induced upon stimulation with MT buffer supplemented with 2.7 mM CaCl_2_ and 10 μM TG in the presence of FPP.

### Immunofluorescence staining and confocal microscopic analysis

Microtubule formation was visualized by immunofluorescence microscopy by the previously described methods (Nugrahini et al. 2020). Briefly, RBL-2H3 cells seeded and pre-cultured in a multi-well bottom dish (#D141400, MATSUNAMI, Osaka, Japan) were sensitized with anti-DNP IgE mAb. Then the cells were treated with FPP or 3 μM nocodazole (a positive control reagent as a microtubule formation inhibitor) for 30 min at 37 °C, followed by 5 min stimulation with 0.5 μg/ml DNP-BSA. After washing with PBS, cells were fixed with 4% formaldehyde/PBS at room temperature for 15 min. Then, the cells were permeabilized with 0.2% Triton X-100/PBS at room temperature for 15 min. For antibody staining, cells were blocked with 5% BSA/PBS for 1 h and stained with Alexa Fluor® 488 conjugated anti-α-Tubulin mAb at 4 °C overnight. After washing and nuclear staining with 0.1 μg/ml DAPI/PBS at room temperature for 30 min, the cells were subjected to anti-α-tubulin immunofluorescence imaging by a LSM700 confocal microscope (Zeiss, Oberkochen, Germany).

### Immunoblot analysis

Immunoblot analysis was performed as described (Kamei et al. 2017). Briefly, anti-DNP IgE-sensitized RBL-2H3 cells were stimulated with 0.5 μg/ml DNP-BSA/MT buffer at 37 °C, and cell samples were collected at 1, 2, 5, and 10 min. The cells were lysed in RIPA buffer (Nacalai tesque) supplemented with phosphatase inhibitor cocktail (Nacalai tesque) upon incubation at 4 °C for 30 min. Soluble cell lysates were obtained via centrifugation at 14,000 × *g* for 15 min. The protein concentration of each cell lysate was determined using the Bio-Rad Protein Assay Reagent (Bio-Rad Laboratories, CA, USA) for equalized protein loading. Resultant protein samples were resolved by sodium dodecyl sulfate–polyacrylamide gel electrophoresis (SDS-PAGE) under reducing conditions, and then electronically transferred onto polyvinylidene fluoride (PVDF) membranes (Immobilon-P; Merck Millipore, Burlington, MA, USA) using the WSE-4040 apparatus (ATTO, Tokyo, Japan). After washing (10 min wash for three times) with tris-buffered saline (150 mM NaCl/50 mM Tris–HCl, pH7.4) supplemented with 0.1% Tween-20 (TBST), the membranes were blocked with 5% skim milk/TBST or 5% BSA/TBST at room temperature for 1 h, followed by overnight incubation with primary antibodies at 4 °C. After washing, membranes were incubated with HRP-conjugated secondary antibodies at room temperature for 1 h. After the final washing, protein bands were visualized using the ECL Prime Western Blotting Detection System (Cytiva, Marlborough, MA, USA). Phosphorylated protein signals were quantified using ImageJ software after normalization to total protein signals.

### Statistical analysis

Data are represented as mean ± standard deviation (SD). Representative data are obtained from at least three independent experiments. Statistical analysis was conducted using a one-way analysis of variance (ANOVA) with a Tukey–Kramer post-hoc test for multiple comparisons. Statistical significance was defined as *P* < *0.05*.

## Results

### FPP suppresses IgE-mediated degranulation of RBL-2H3 rat basophilic leukemia cells

**Fig. 1:**
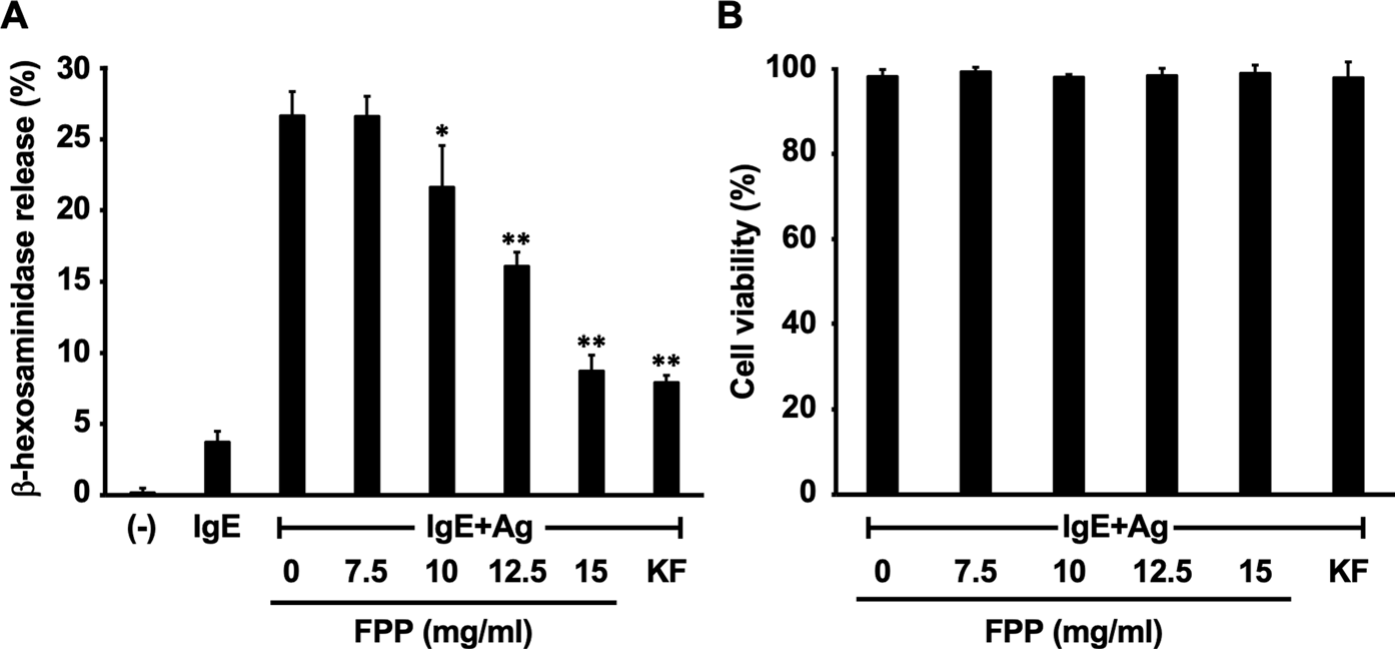
FPP suppresses IgE-mediated mast cell degranulation. **A**, Stimulation with FPP suppresses degranulation of RBL-2H3 cells. Degranulation was evaluated by the enzymatic activity of released β-hexosaminidase from IgE-sensitized RBL-2H3 cells upon pre-stimulation with a serial dose of FPP for 30 min, followed by stimulation with antigen (Ag; DNP-BSA) for 30 min. Ketotifen fumarate (KF, 500 μM) was used as a positive control for the inhibition of degranulation. *P < *0.05*; **P < *0.01 vs* “IgE + Ag without FPP “. **B**, FPP has no inhibitory effect on the cell viability of IgE-sensitized RBL-2H3 cells. RBL-2H3 cells were cultured with a serial dose of FPP or KF for 30 min, and stimulation with Ag for 30 min. Cell viability was then analyzed by trypan blue dye exclusion assay

A previous study has shown that oral FPP supplementation suppresses allergic rhinitis-like nasal symptoms in a murine model of Japanese cedar pollinosis, while allergen-specific IgE and T cell responses are unaffected upon feeding with FPP (Fujimura et al. 2018). In this system, oral FPP supplementation down-regulates serum level of mouse mast cell protease-1 (mMCP-1), an in vivo biomarker of mast cell-mediated allergic inflammation (Fujimura et al. 2018). This prompted us to assess the possibility that FPP acts on the mast cells to suppress immediate hypersensitivity reactions. To test this assumption, we first tested whether FPP suppressed IgE-mediated mast cell degranulation in vitro. We found that co-stimulation with FPP significantly suppressed DNP-BSA antigen (Ag)-driven β-hexosaminidase release from anti-DNP-IgE-sensitized RBL-2H3 rat basophilic leukemia cells in a dose-dependent manner (Fig. 1A). The IC_50_ value for the degranulation inhibitory activity of water-soluble FPP fraction as 13.6 mg/ml. We also confirmed that the in vitro FPP stimulation did not show cytotoxicity against RBL-2H3 cells, as revealed by trypan blue dye exclusion assay (Fig. 1B). These results suggest that FPP acts directly on mast cells to suppress IgE-mediated degranulation.

### Oral feeding with FPP suppresses in vivo immediate hypersensitivity reactions

**Fig. 2:**
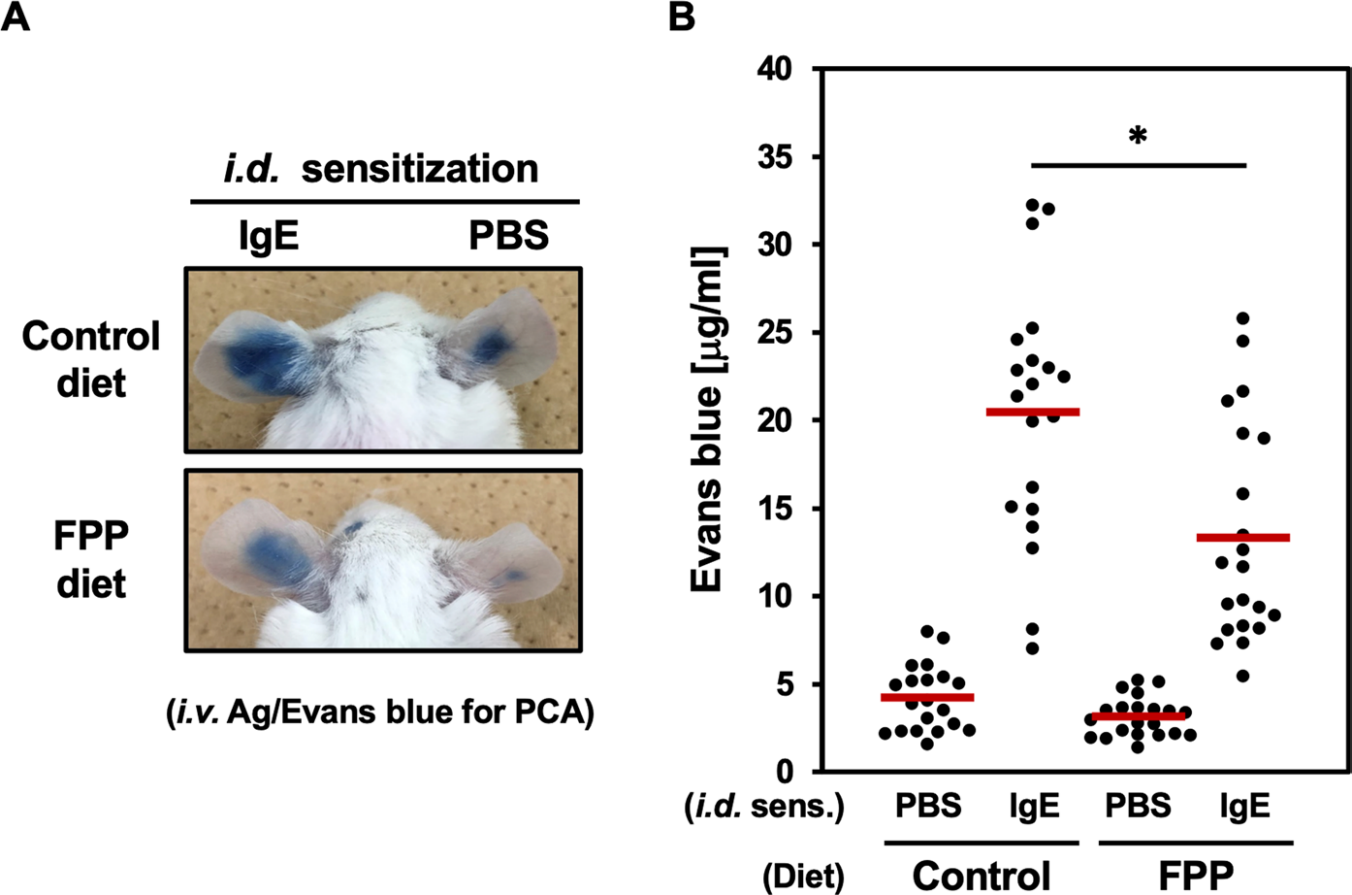
Oral administration of FPP suppresses PCA. **A**, Representative photographs of the auricles in which PCA induction in the left ears was visualized by extravasation of Evans blue dye. lefts, intradermal (*i.d.*) sensitization with anti-DNP IgE mAb; rights, *i.d.* sensitization with PBS as a negative control. **B**, FPP supplementation significantly suppresses PCA. Extravasation of Evans blue dye was quantified by spectrophotometry. *P < *0.05*

We next tested whether FPP also suppresses in vivo immediate hypersensitivity reaction by its oral feeding to a PCA model mouse. The oral FPP supplementation significantly suppressed PCA, as indicated by impaired extravasation of Evans blue dye in the IgE-sensitized ears (Fig. 2A and B). These results clearly indicate that FPP attenuates IgE and mast cell-mediated immediate hypersensitivity reactions in vivo.

### FPP suppresses FcεRI ligation-driven intracellular Ca^2+^ influx and microtubule formation, both of which are essential for provoking IgE-mediated mast cell degranulation

**Fig. 3:**
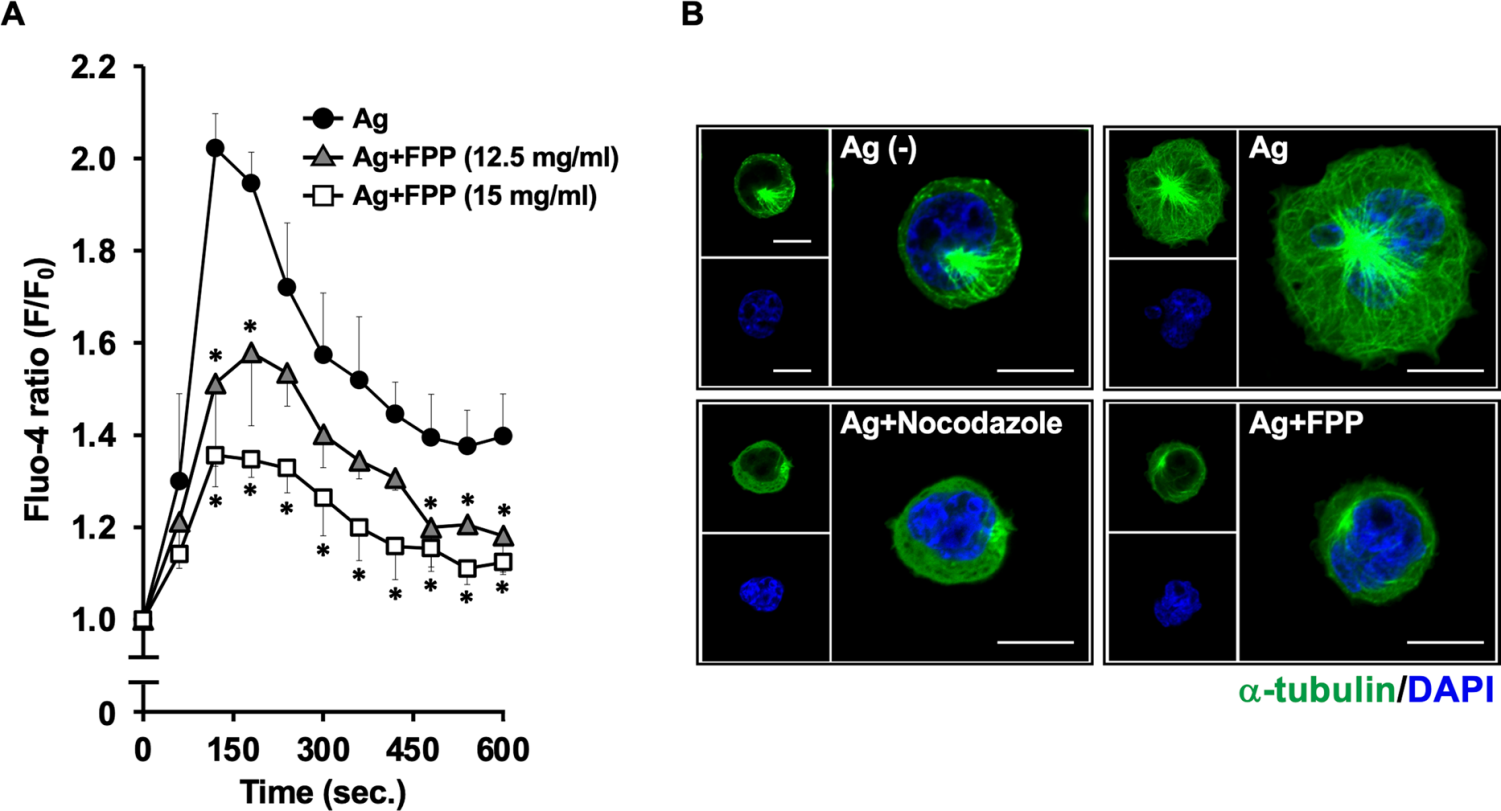
FPP suppresses FcεRI-driven intracellular Ca^2+^ influx and microtubule formation, both of which are essential for IgE-mediated degranulation of mast cells. **A**: Stimulation with FPP inhibits intracellular Ca^2+^ influx. Ca^2+^ influx was monitored using Fluo 4-AM, a Ca^2+^-sensitive fluorescence probe. Fluo 4-AM loaded, and IgE-sensitized RBL-2H3 cells were treated with a serial dose of FPP for 30 min, followed by stimulation with Ag (DNP-BSA), and then immediately subjected to time-course analysis of Ca^2+^ mobilization. The vertical axis was represented as relative changes in Fluo-4 emission (F/F_0_). *P < *0.05 vs* “Ag” (without FPP). **B**, FPP suppresses microtubule formation upon FcεRI cross-linking. IgE-sensitized RBL-2H3 cells were pre-treated with 15 mg/ml FPP or 3 μM nocodazole (a microtubule inhibitor, as a positive control) for 30 min, followed by stimulation with Ag for 5 min. Then the cell samples were co-stained with Alexa Fluor 488-conjugated anti-α-tubulin mAb (green) and DAPI (blue). Immunofluorescence signal was visualized by confocal laser scanning microscopy. The scale bar shows 10 μm

We next sought to elucidate mechanisms by which FPP suppresses immediate hypersensitivity reactions. The IgE-mediated mast cell degranulation is triggered through antigen-driven cross-linking of IgE, which is pre-sensitized on the mast cells via high-affinity IgE receptor, FcεRI (Blank et al. 2021) (Kawakami and Galli 2002). The FcεRI ligation activates two signaling events essential for mast cell degranulation; one is intracellular Ca^2+^ influx needed for granule exocytosis (Nadler and Kinet 2002) (Blank and Rivera 2004) (Huber et al. 2019), and another is a Ca^2+^-independent microtubule formation that is critical for granule translocation to the plasma membrane (Nishida et al. 2005) (Ogawa et al. 2014). We first assessed whether FPP shows an inhibitory effect on those two FcεRI signaling pathways in RBL-2H3 cells. We found that treatment with FPP significantly suppressed the FcεRI ligation-driven intracellular Ca^2+^ influx (as indicated by increased Fluo-4 Ca^2+^ indicator signal) in a dose-dependent manner (Fig. 3A). We also found that the FPP stimulation inhibited the Ca^2+^-independent microtubule formation upon FcεRI ligation, as demonstrated by immunofluorescent staining of α-tubulin (Fig. 3B). These results indicate that FPP suppresses FcεRI ligation-driven intracellular Ca^2+^ influx and microtubule formation, both of which are essential for IgE-mediated mast cell degranulation.

### FPP extensively suppresses the Ca^2+^-dependent- and -independent FcεRI signaling pathways

**Fig. 4:**
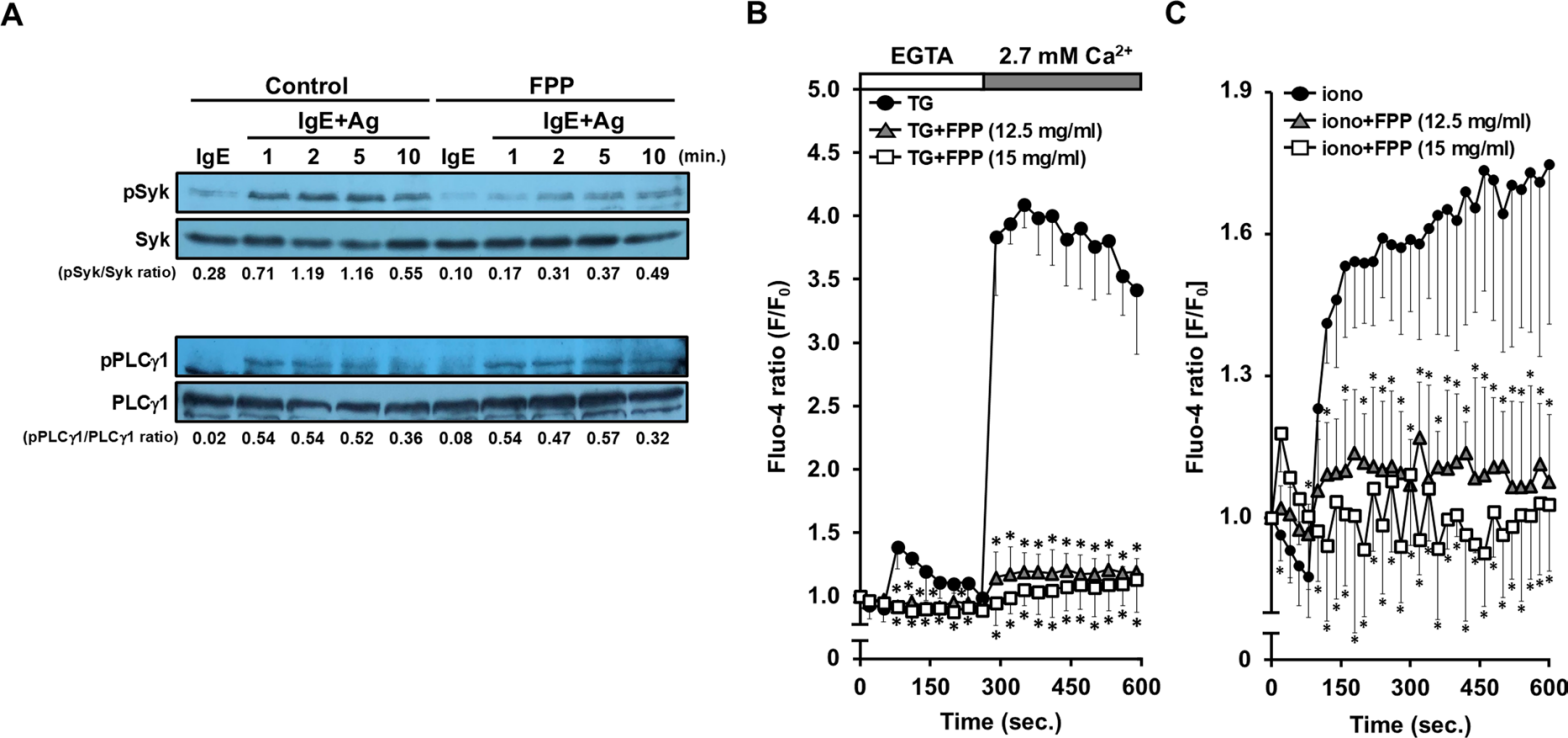
FPP suppresses Ca^2+^-dependent FcεRI signaling pathway. **A**, Effect of FPP on the activation of the Syk-PLCγ1 axis upon FcεRI cross-linking. IgE-sensitized RBL-2H3 cells were treated with 15 mg/ml FPP for 30 min, followed by Ag (DNP-BSA) stimulation for 1, 2, 5 and 10 min. Each cell lysate was subjected to immunoblot analysis with specific antibodies against Syk, PLCγ1, or their phosphorylated forms. **B**, FPP inhibits thapsigargin (TG)-induced store-operated calcium entry (SOCE). Fluo 4-AM loaded RBL-2H3 cells were pre-treated with a serial dose of FPP for 30 min in Ca^2+^-free medium, and then stimulated with 10 μM TG. The cells were immediately subjected to a time-course analysis of Ca^2+^ mobilization upon addition of TG. The vertical axis indicates relative changes in Fluo-4 emission (F/F_0_). *P < *0.05 vs* “TG” (Thapsigargin without FPP). **C**, FPP inhibits ionomycin-induced SOCE. Fluo 4-AM loaded, and FPP-pre-treated RBL-2H3 cells were stimulated with 0.9 μM ionomycin. The vertical axis indicates relative changes in Fluo-4 emission (F/F_0_). *P < *0.05 vs* “iono” (ionomycin without FPP)

**Fig. 5:**
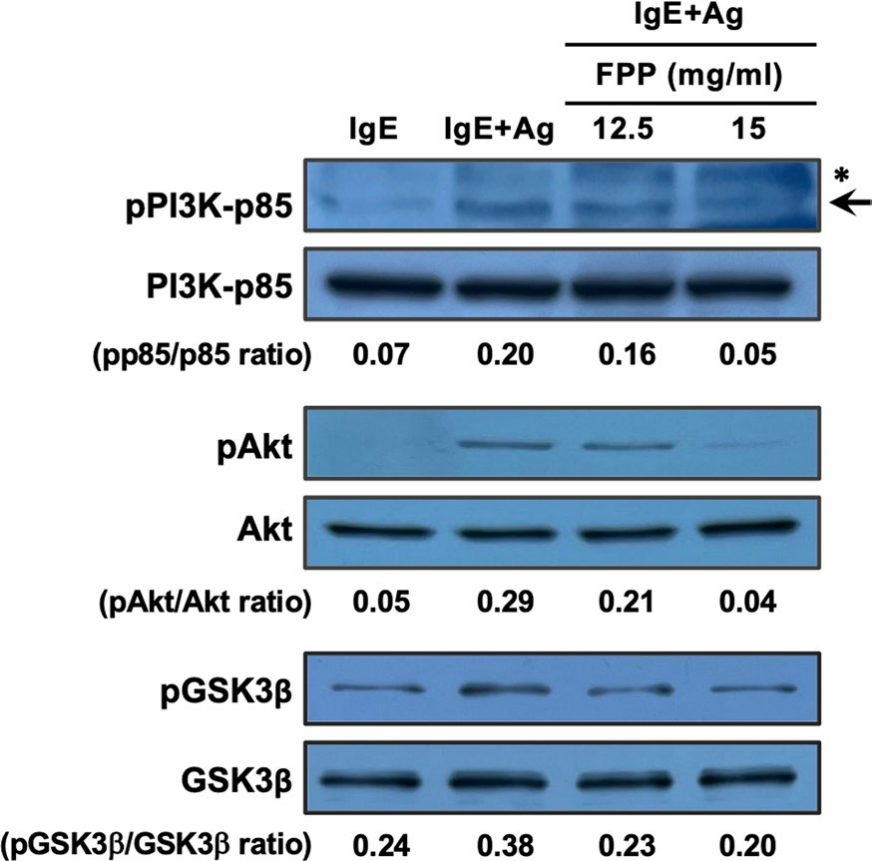
FPP negatively regulates the Ca^2+^-independent FcεRI signaling pathway. FPP suppresses activation of the PI3K-Akt-GSK3β axis upon FcεRI cross-linking. IgE-sensitized RBL-2H3 cells were pre-treated with 15 mg/ml FPP for 30 min, followed by stimulation with Ag (DNP-BSA). Cell lysates were subjected to immunoblot analysis. An arrow indicates anti-phospho-PI3K-p85 (pPI3K-p85)-specific signals, and asterisk indicates non-specific bands

We further analyzed detailed molecular mechanisms by which FPP negatively regulates the FcεRI signaling pathways to suppress the degranulation of RBL-2H3 cells. To clarify the suppressive mechanisms of FPP on intracellular Ca^2+^ influx, we tested the effect of FPP stimulation on the phosphorylation of Syk and PLCγ1, upstream molecules of the Ca^2+^-dependent FcεRI signaling pathway. We found that FPP stimulation seemed to inhibit the phosphorylation of Syk upon FcεRI ligation, while phosphorylation of PLCγ1 was unaffected (Fig. 4A), suggesting that FPP acts on molecular events downstream of PLCγ1 to suppress Ca^2+^ influx. We next tested whether FPP inhibited the downstream store-operated calcium entry (SOCE) to suppress intracellular Ca^2+^ influx. As shown in Fig. 4B, FPP strikingly suppressed SOCE upon pharmacological treatment with thapsigargin, a　sarcoplasmic/endoplasmic reticulum Ca^2+^ ATPase (SERCA) inhibitor that induces SOCE via Ca^2+^ depletion from the endoplasmic reticulum (ER) (Thastrup et al. 1990). Another pharmacological evidence further confirmed that FPP also suppressed the low-dose (0.9 μM) ionomycin-induced SOCE (Morgan and Jacob 1994) (Fig. 4C). These results suggest that FPP suppresses the Ca^2+^-dependent FcεRI signaling pathway at least in part by down-regulation of the SOCE processes. We also explored the mechanisms by which FPP negatively regulates another Ca^2+^-independent FcεRI signaling pathway (the PI3K-Akt-GSK-3β pathway), leading to microtubule formation (Ogawa et al. 2014). We found that FPP suppresses the FcεRI ligation-driven phosphorylation of PI3K, Akt, and GSK-3β (Fig. 5). Taken together, these mechanistic analyses suggest that FPP extensively suppresses both the Ca^2+^-dependent- and -independent FcεRI signaling pathways to inhibit immediate hypersensitivity reactions.

## Discussion

The previous study has shown that oral FPP supplementation prevents the development of allergic rhinitis-like nasal symptoms without affecting systemic immune response, suggesting that FPP may act on the IgE-mast cell axis to exert its anti-allergic effect (Fujimura et al. 2018). Here, we provide evidence that FPP suppresses IgE-and mast cell-mediated type I hypersensitivity reactions in vitro as well as in vivo, and that the potent anti-allergic effect of FPP is fulfilled by extensive negative regulation of the FcεRI signaling pathway.

The canonical FcεRI signaling branches into Ca^2+^-dependent, and Ca^2+^-independent pathways, and activation of both two pathways is critical for mast cell degranulation upon FcεRI ligation (Nishida et al. 2005) (Ogawa et al. 2014). Our data indicate that FPP potently suppresses both two pathways, as evidenced by inhibition of FcεRI-mediated intracellular Ca^2+^ influx (Fig. 3A) and Ca^2+^-independent microtubule formation (Fig. 3B). The dual inhibitory effect of FPP on both Ca^2^⁺-dependent and Ca^2^⁺-independent FcεRI signaling provides a molecular basis for its potent anti-allergic activity, as evidenced by its oral administration significantly prevents immediate hypersensitivity reactions (Fig. 2) and Japanese cedar pollinosis (Fujimura et al. 2018). How does FPP negatively regulate these two pathways simultaneously? Especially in the Ca^2+^-independent pathway on which FPP fully suppresses the corresponding PI3K-Akt-GSK-3β signaling axis (shown in Fig. 5), one possible upstream target of FPP could be Syk, whose activation (tyrosine phosphorylation) is suppressed upon stimulation with FPP (Fig. 4A). In agreement with this assumption, Kitaura et al*.* (Kitaura et al. 2000) and Barbu et al*.* (Barbu et al. 2010) also have demonstrated that Syk works as a critical upstream activator of the PI3K-Akt pathway in mast cells.

Our mechanistic analysis on the Ca^2+^-dependent FcεRI signaling pathway revealed that FPP directly suppresses SOCE (Fig. 4B and C) to inhibit intracellular Ca^2+^ influx. Intriguingly, FPP not only inhibits SOCE provoked by a SERCA inhibitor thapsigargin (in the presence of Ca^2+^ ions, as shown in Fig. 4B), but also abolishes another thapsigargin-induced first Ca^2+^ spike, a sign of Ca^2+^ depletion from the ER (under Ca^2+^-free conditions, shown in Fig. 4B). This observation suggests that FPP antagonizes SERCA inhibitory activity of thapsigargin, which leads to impaired Ca^2+^ depletion from the ER to suppress SOCE. In this scenario, the most plausible mechanism is that FPP may up-modulates Ca^2+^ ATPase activity of SERCA to facilitate Ca^2+^ entry into the ER (*i.e.*, suppression of Ca^2+^ depletion from the ER), which in turn blocks downstream SOCE machinery to suppress intracellular Ca^2+^ influx. Indeed, a recent pre-clinical trial indicates that activation of SERCA represents a new anti-allergic strategy to suppress IgE-mediated mast cell activation (Hunter et al. 2023). Further analysis is needed to see whether FPP activates SERCA to suppress Ca^2+^ influx upon FcεRI cross-linking. The potential SERCA activation capacity of FPP may provide a mechanistic explanation for how FPP suppresses the Ca^2^⁺-dependent FcεRI signaling pathway while leaving upstream PLCγ1 activity unaffected (Fig. 4A). The FPP-mediated inhibition of SOCE (Fig. 4B and C) through SERCA activation stabilizes ER Ca^2^⁺ levels and inositol 1,4,5-trisphosphate (IP₃) receptor-dependent Ca^2^⁺ influx. This sustained Ca^2^⁺ signaling may enhance the Ca^2^⁺-dependent feedback activation of PLCγ1 (Thore et al. 2005).

The molecular nature of anti-allergic components in FPP needs further elucidation. Candidate active compounds could be pre-existing compounds from starter plant materials, or their fermented products. Another possible case is that those multiple compounds act synergistically to exert the mast cell-targeted anti-allergic action of FPP. Fruits and citrus contain abundant polyphenols that have been reported to show anti-allergic effects acting on mast cells (Debinska and Sozanska 2023). In fact, the most abundant starter materials for manufacturing FPP are fruits (*e.g.* apple, persimmon), and citrus accounts for 40.1% of total starter materials (Kawai 1994). Thus, those fruits-derived phytochemicals can be responsible for anti-allergic compounds in FPP. Another interesting study has demonstrated that sake lees (a byproduct of Japanese sake fermentation) fermented with lactic acid bacteria suppresses allergen-induced mast cell degranulation and allergic rhinitis-like nasal symptoms, while control unfermented sake lees starter fails to show such anti-allergic activity (Kawamoto et al. 2011). This suggests that de novo-synthesized compounds through fermentation, but not those from starter raw plant materials, can also be responsible for the anti-allergic effect of fermented sake lees. Our preliminary observations indicate that the anti-allergic components of FPP passed through the 10 kDa ultrafiltration filter (T. Kodama and S. Kawamoto, unpublished data). Further analysis is now underway to identify the active compounds in the FPP. The IC_50_ value for the degranulation inhibitory activity of water-soluble FPP fraction as 13.6 mg/ml (Fig. 1A). We also tested the degranulation inhibitory activity of whole FPP containing insoluble fractions and found that the entire FPP potently suppressed the degranulation of RBL-2H3 cells with the IC_50_ value of 11.4 mg/ml, which seems to be comparable to that of water-soluble FPP fraction (Kodama and Kawamoto, unpublished data). These data suggest that the soluble fraction of FPP mainly accounts for its anti-allergic potency, while insoluble components may also be involved in the in vivo PCA suppressive potency of dietary FPP containing insoluble ingredients.

## Conclusions

In the present study, we show that FPP potently suppresses IgE- and mast cell-mediated immediate hypersensitivity reactions in vitro as well as in vivo. We also provide mechanistic evidence that FPP negatively regulates FcεRI signaling pathway to suppress mast cell degranulation. The present study highlights the potential utility of dietary FPP supplementation in the prevention and/or attenuation of immediate hypersensitivity reactions targeting mast cell degranulation.

## Data Availability

No datasets were generated or analysed during the current study.

## Abbreviations

FPP: Fermented plant product
Th2: T helper type 2
mMCP-1: Mouse mast cell protease-1
MT Buffer: Modified Tyrode’s buffer
BSA: Bovine serum albumin
MEM: Minimum essential medium
FBS: Fetal bovine serum
KF: Ketotifen fumarate
Ag: Antigen, PCA: Passive cutaneous anaphylaxis
HSA: Human serum albumin
EB: Evans blue
SOCE: Store-operated Ca^2+^ entry
EGTA: Ethylene glycol tetraacetic acid
TG: Thapsigargin
ER: Endoplasmic reticulum
SDS-PAGE: Sodium dodecyl sulfate–polyacrylamide gel electrophoresis
PVDF: Polyvinylidene fluoride
TBS: Tris-buffered saline
SD: Standard deviation
ANOVA: Analysis of variance
*i.d.*: Intradermal
SERCA: Sarcoplasmic/endoplasmic reticulum Ca^2+^ ATPase
